# Collar-button abscess as a complication of infected hematoma in the setting of uncontrolled type 2 diabetes

**DOI:** 10.1016/j.ijscr.2022.107427

**Published:** 2022-07-20

**Authors:** Jennifer Adams, Daniel Habenicht, Savvas Poulos

**Affiliations:** a University of Texas Rio Grande Valley, School of Medicine, 1201 W University Dr., Edinburg, TX, USA; b Department of Orthopedic Surgery, Valley Baptist Medical Center, Harlingen, TX, USA

**Keywords:** Collar-button abscess, Diabetes, Hand infections

## Abstract

**Introduction:**

Collar-button abscesses are deep space infections of the hand.

**Case presentation:**

We present a case of a 66-year-old man who developed an acute collar-button abscess of the hand after a concrete bench fell onto the dorsal aspect of his hand. The hand abscess was managed successfully with intravenous antibiotics and operative intervention.

**Discussion:**

While such infections comprise a small percentage of hand infections, insufficient or delayed treatment results in permanent hand disfiguration and dysfunction. This case highlights an uncommon dorsal-to-volar pattern of hand abscess extension.

**Conclusion:**

Knowledge of the anatomy of the hand is essential to diagnosis and appropriate surgical management.

## Highlights

• We present a collar-button hand abscess that began as a dorsal web space infection with quick invasion into the volar space.
• Collar button abscesses may mimic the presentation of cellulitis and suppurative tenosynovitis.
• Diabetic hand infections are poorly understood and recognized, often resulting in delayed diagnosis and severe morbidity.

## 1 Introduction

Diabetic hand-related infections are poorly understood and less commonly recognized compared to foot-related infections, which often result in delayed diagnosis and severe morbidity and mortality. The incidence of deep space hand infections is higher in patients with poorly controlled diabetes [1]. A collar-button abscess is one complication of a web space infection involving both palmar and dorsal hand. The diagnosis is clinical, and suspicion should be raised when an abscess forms at the distal edge of the palm in the spaces separating adjacent fingers [2].

The most common infectious agents are *Staphylococcus aureus* and *group A Streptococcus*, with *S. aureus* reported to be present in 80 % cultures [3]. The choice of surgical technique is important when treating a collar button abscess. Because the abscess is hourglass-shaped extending through the transverse plane, both volar and dorsal incisions are required [2].

We present an unusual case of an aggressive collar-button hand abscess that began as an infection of the dorsal web space with later invasion into volar space. We achieved a favorable resolution with aggressive surgical treatment. This case highlights the importance of early detection and expedited treatment since clinical presentation is often overlooked until serious consequences occur.

This work has been reported in line with the SCARE 2020 criteria [10].

## 2 Case presentation

**Fig. 1:**
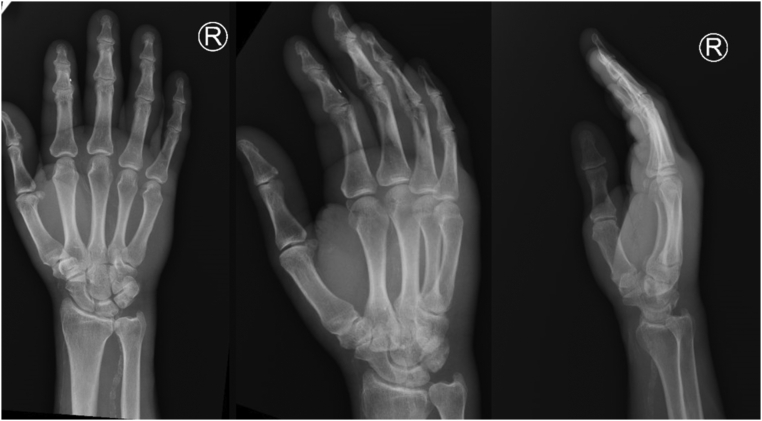
Right hand radiographs showing soft tissue swelling extending to the fourth digit and normal bone anatomy (from left to right: AP, Oblique, Lateral views).

A 66-year-old man from Mexico presented to the hospital with a one-week history of worsening right hand pain, swelling and erythema most prominent in the distal palm. Physical examination demonstrated a painful 1.5-cm fluctuant mass extending from the palmar to the A2 pulley of the right fourth digit, with focal tenderness and swelling over the flexor sheath and limited digital motion. Patient was started on vancomycin and piperacillin-tazobactam. Patient denied the use of any medications. His medical history was notable for type 2 diabetes mellitus with HbA1c of 11.2 %. He reported a history of blunt injury to the base of the fourth digit on the dorsal aspect of the right hand after a concrete bench fell on his hand two weeks from the time of presentation. At the time of the fall, he did not observe any lacerations or breaks in the skin. Over the subsequent week, he reported observing a bruise forming over the site of injury. Blood tests were significant for white cell count of 17.5 × 10^9^/L. Radiographs of the hand revealed soft tissue swelling in entire right palm, extending to the distal interphalangeal joints of the third and fourth digits with intact bony anatomy, without fractures or dislocations (Fig. 1).

The in-house hand surgeon performed the procedure. After informed consent was obtained, the patient underwent incision, drainage, irrigation under general anesthesia. On inspection, there was a large blister on the dorsum of the finger with a cloudy fluid. On incision, this blister was opened and then an incision was made in the fourth digit of the right hand and extended distally between the metacarpal area. Immediate purulent fluid was expressed and samples were collected for culture. Further spreading was performed extending proximally to break up loculations. Purulence was removed, and the area was irrigated. Care was taken to visualize and protect neurovascular bundles. After gentle spreading performed to ensure no other pockets of purulence was present, attention was turned to the volar aspect of the finger. An oblique zigzag-type incision was performed over the A1 pulley region, the area of most fluctuance dorsally. Purulence was noted on the volar aspect indicative of infection spreading from distal to proximal consistent with a collar button abscess in the deep space between the volar and extensor area. This area was cleared and irrigated. Further spreading was performed down to the sheath and then a slight opening was made in the flexor sheath proximally. No purulence was noted in the flexor sheath and flexor tendons were loosely intact with preservation of passive flexion and extension. After further irrigation and ensuring absence of additional purulent pockets, packing was applied and a drain was placed through the webspace. The wound was packed volarly and then the skin edges were approximated with a nylon suture.

**Fig. 2:**
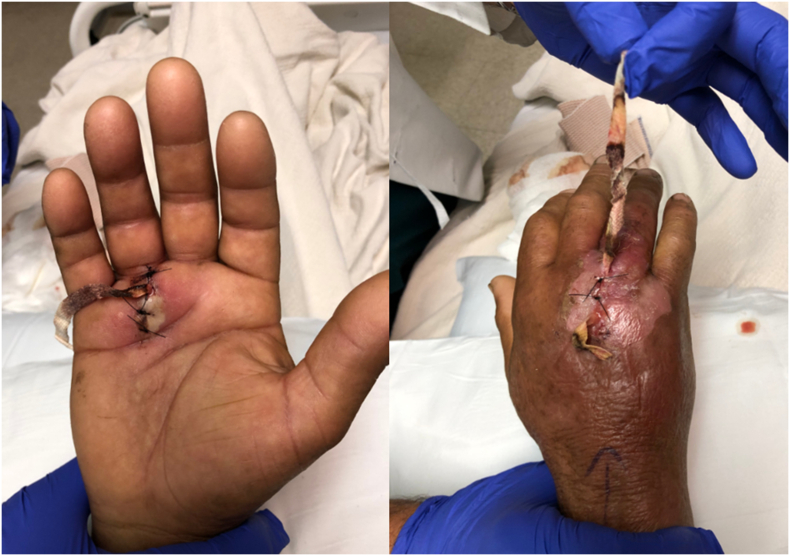
Photograph of right hand on postoperative day 2.

Intraoperative cultures identified the growth of Methicillin-sensitive *Staphylococcus aureus*. The patient returned to the operating room after 48 h for a second look operation (Fig. 2). An area of fatty necrosis with purulent fluid in the dorsal wound was visualized. The volar wound had a slight expression of cloudy fluid on the ulnar aspect, but the tendon sheath was noted to be intact with no purulence or exudate within the tendon sheath at the A1 pulley region. The wound were thoroughly irrigated and necrotic tissue was debrided. The drain was removed 48 h after the second debridement. Postoperatively, his symptoms improved significantly, and he was discharged home on oral doxycycline for ten days and referred to physical therapy.

## 3 Discussion

Collar-button abscess describes a subfascial infection of a web space that tends to spread peripherally at the palmar and dorsal ends and remains narrow in the middle [4]. The shape of the abscess can be described as an “hourglass.” It often occurs via direct inoculation or secondary extension from the adjacent anatomic structures [2]. The diagnosis is clinical and based on an abscess forming at the distal edge of the palm separating the adjacent fingers.

Because of the rigidity of the palmar aponeurosis and the adherence of the palmar skin and underlying fascia, the infection has the tendency to expand dorsally through the space in the palmar fascia to involve the subcutaneous tissue of the dorsal web [1]. The borders of the midpalmar space include the flexor tendons of the ulnar three digits, hypothenar septum, and interosseous muscles. An abscess in this space will manifest as pain with movement of the middle and ring fingers, which is consistent with our patient's presenting symptoms. The infection forms a tract from the palmar fascia over the superficial transverse metacarpal ligament between the metacarpals to involve the dorsal subcutaneous space. As the infection travels dorsally through the fascial hole, the involved web space becomes swollen and inflamed, which results in abduction of adjacent digits [2], [3].

Deep space hand infection occurs with higher incidence in diabetics. The pathophysiology has not been clearly investigated but is thought to involve advanced glycated end-products (AGES) and microangiopathy of the distal extremities [4]. Many patients with diabetes develop changes in the connective tissues of the hands. Diabetic cheiroarthropathy is a documented clinical entity in which the skin and underlying tissues of the hand become thickened, resulting in a limited range of movement of the hand and finger joints. Biopsy often demonstrates excessive fibrosis and increased deposition of dermal collagen [5]. It has been suggested that increased tissue glycation may result in diminished collagen breakdown, predisposing to the weakening and contraction of fascia in hand. Fascial compromise increases susceptible to infection spread and communication between facial spaces [6]. This mechanical weakening within the anatomy combined with immunocompromised status and impaired perfusion and nerve function further predispose diabetic patients to rapid spread of infection and a poor tendency to heal.

Collar button abscess may present similarly to cellulitis and suppurative infections. Our patient's condition was initially misdiagnosed as cellulitis and fourth digit tenosynovitis. Flexor tendon sheath infections are devastating and are clinically recognized by Kanavel's four cardinal signs: tenderness along the course of the flexor tendon sheath, semiflexed digit positioning, pain with passive extension of the digit, and symmetric swelling of the entire digit [7]. Our patient had two out of the four Kanavel signs on presentation, with the absence of tenderness along entire course of the fourth flexor tendon sheath and fusiform swelling, leading to initial concerns for cellulitis and flexor tenosynovitis. It is important to include deep space abscess in the differential diagnosis for future cases similar to this one because clinical and laboratory indicators are often variably present. Delayed identification and management can result in poor outcomes with permanent deficits.

Proper decompression of a collar button abscess always requires volar and dorsal incisions because of the communicating channel between the volar and dorsal hand [1]. Many incision patterns exist, but treatment should involve two incisions, a longitudinal incision between digits on the dorsal hand and a volar Z-pattern incision [8], [9]. These incisions should extend between the proximal edge of the web and distal palmar crease to avoid the common digital neurovascular bundle.

## 4 Conclusion

This patient had a bruise on the dorsal aspect of the hand, which we hypothesize to have become infected and involve deeper fascial spaces. This case is unique in that the abscess propagated by dorsal to palmar spread. Collar button abscesses usually result from contiguous spread dorsally, most often from an infected palmar injury. A high index of suspicion coupled with excellent knowledge of hand anatomy and function allows for accurate diagnosis and effective management. It is important to diagnose collar-button abscess early as it guides the decision to make two incisions, one on dorsal and another on volar aspect of the hand, to avoid incomplete evacuation of abscess and reoperation. Delay in treatment could result in the spread of infection throughout the deep spaces, risking significant compromise of hand function and damage to neurovascular structures.

## Patient perspective

N/A.

## Provenance and peer review

Not commissioned, externally peer-reviewed.

## Sources of funding

None.

## Ethical approval

Not applicable.

## Consent

Written informed consent was obtained from the patient for publication of this case report and accompanying images. A copy of the written consent is available for review by the Editorin-Chief of this journal on request.

## Author contribution

JA and DH were equally involved in the writing of the manuscript. SP was involved in the editing/supervision of the manuscript.

We would like to acknowledge Dr. Antonio Alvarez-Mendoza for his insights, guidance and expertise with the pathological specimens from this case.

## Research registration

N/A.

## Guarantor

Savvas Poulos MD.

## Declaration of competing interest

All authors declare no conflicts of interest.
